# Lower-Extremity CT Angiography: Inter-Hospital Dose Variability and Protocol Optimization in Sudan

**DOI:** 10.7759/cureus.101678

**Published:** 2026-01-16

**Authors:** Nada A Ahmed, Leena A Ali

**Affiliations:** 1 Department of Physics, College of Science, Taibah University, Madinah, SAU; 2 Department of Radiology, Radiation Protection Centre, Emirates Health Service, Dubai, ARE

**Keywords:** ctdi vol, dlp, lower extremity cta, protocol optimization, radiation dose

## Abstract

Introduction

Lower-extremity CT angiography (CTA) is widely used for the evaluation of peripheral arterial disease but is associated with substantial radiation exposure. Even when identical CT scanners are used, variability in protocol design may lead to marked differences in patient dose.

Purpose

The primary aims of this study are: assess inter-hospital variability in radiation dose for lower-extremity CTA performed on identical 64-slice CT scanners (Aquilion 64; Toshiba Medical Systems, Otawara, Japan), identify protocol-related factors amenable to dose optimization within existing CT system capabilities.

Methods

This multicentre retrospective observational study included 74 adult patients undergoing clinically indicated lower-extremity CT angiography at four hospitals using identical 64-slice CT scanners (Hospital one: n=15; Hospital two: n=21; Hospital three: n=17; Hospital four: n=21). Radiation dose metrics, including volume CT dose index (CTDI_(vol)_) and dose-length product (DLP), were collected for all scan phases performed, including the localizer, bolus-tracking, non-contrast helical phase (when clinically indicated), and contrast-enhanced helical acquisitions. No BMI or weight-based exclusion criteria were applied. Phase-specific and cumulative dose metrics were analyzed in relation to acquisition parameters and scan coverage.

Results

Mean cumulative DLP ranged from 5333.2 to 7911.6 mGy·cm across centres, corresponding to an approximately 1.5-fold difference. Non-contrast and contrast-enhanced helical phases were the dominant contributors to total radiation exposure, with phase-specific DLP values frequently exceeding 1000 mGy·cm. In centres performing non-contrast imaging, this phase occasionally contributed a higher DLP than the contrast-enhanced phase, indicating that selective omission could reduce cumulative dose. In other centres, elevated CTDI_(vol)_ during contrast-enhanced and bolus-tracking phases offset dose reductions achieved through shorter scan lengths. Several protocols exceeded published regional and international diagnostic reference levels. Published evidence supports that low-tube-voltage acquisition (70-80 kVp) combined with iterative reconstruction can substantially reduce radiation dose and represents a feasible optimization strategy within current scanner capabilities.

Conclusion

Radiation dose in lower-extremity CTA is largely determined by protocol design rather than scanner hardware. Strategies including selective non-contrast phase omission, low-tube-voltage acquisition, iterative reconstruction, and optimized bolus-tracking can substantially reduce dose while maintaining diagnostic quality. Harmonized implementation across centres is essential for consistent and meaningful dose reduction.

## Introduction

Computed tomography (CT) is a cornerstone of modern diagnostic imaging, offering high spatial and contrast resolution that enables rapid, non-invasive evaluation across a wide range of clinical conditions. Technological advances, including faster gantry rotation, wider detector coverage, and iterative reconstruction techniques, have further expanded its clinical applications [1,2]. However, the widespread and increasing use of CT has resulted in a substantial rise in population exposure to medical ionizing radiation. According to the United Nations Scientific Committee on the Effects of Atomic Radiation (UNSCEAR), CT contributes a disproportionately large share of the collective effective dose from diagnostic radiology, despite accounting for a smaller proportion of total imaging procedures [3].

This contribution is particularly pronounced in complex, multiphase examinations such as CT angiography (CTA), where multiple sequential acquisitions are often required to achieve optimal vascular visualization and diagnostic confidence [1,2]. Lower-extremity CTA is widely used for the evaluation of peripheral arterial disease (PAD), providing detailed anatomical information essential for clinical decision-making and procedural planning [4,5]. Numerous studies have demonstrated that CTA achieves diagnostic accuracy comparable to digital subtraction angiography while being less invasive and more accessible [5].

Despite these advantages, lower-extremity CTA is associated with relatively high radiation dose, primarily due to extended scan coverage from the abdominal aorta to the pedal arteries and the frequent use of multiphase acquisition protocols. A typical CTA examination may include planning images, bolus-tracking sequences, non-contrast imaging, and one or more contrast-enhanced helical phases. Non-contrast imaging is not routinely required for lower-extremity CTA and is therefore selectively employed only in specific clinical situations, such as suspected acute aortic syndromes or active bleeding [6]. Delayed-phase imaging may be performed to provide a second opportunity for optimal arterial visualization of the infrapopliteal vasculature [7]. In addition, bolus-timing monitoring scans repetitively expose the same anatomical region to radiation to determine the appropriate contrast-enhancement threshold [8]. Consequently, careful justification of each acquisition phase, together with optimization of acquisition parameters, is essential to minimize cumulative radiation exposure while preserving diagnostic image quality [1,2].

Published literature demonstrates wide variability in radiation dose associated with lower-extremity CTA, with reported dose-length product (DLP) values ranging from a few hundred to several thousand mGy·cm and CTDI_₍vol₎ _values ranging from approximately 2 to 25 mGy [4,9]. This variability reflects differences in acquisition parameters, scan length, phase selection, and reconstruction techniques rather than scanner technology alone. Although advanced technologies such as dual-source CT systems have demonstrated substantial dose reductions through high-pitch acquisitions and low-kVp imaging, such systems are not universally available, particularly in low-resource settings.

International organizations, including the International Commission on Radiological Protection (ICRP) and the International Atomic Energy Agency (IAEA), emphasize the principles of justification and optimization, advocating adherence to the “as low as reasonably achievable” (ALARA) concept while maintaining diagnostic image quality [1,2]. In many low- and middle-income countries, including Sudan, the absence of established diagnostic reference levels (DRLs) and routine dose auditing may further exacerbate inter-institutional dose variability. To date, lower-extremity CTA has not been systematically evaluated in Sudan, despite its increasing clinical use and inherent potential for high radiation exposure.

Accordingly, the aim of this multicentre study was to quantify patient radiation doses during lower-extremity CTA performed on identical 64-slice CT scanners across multiple hospitals in Sudan and to identify protocol-related factors contributing to inter-hospital dose variability. While acknowledging that advanced scanner technologies can achieve lower radiation doses, this study specifically focuses on identifying practical opportunities for dose optimization within existing CT systems through analysis of phase-specific and cumulative dose metrics, in line with current international radiation protection guidance.

## Materials and methods

This multicentre retrospective observational study was conducted across four hospitals in Sudan performing clinically indicated lower-extremity CT angiography (CTA). All participating centres were equipped with identical Toshiba Aquilion 64-slice multidetector CT (MDCT) scanners (Aquilion 64; Toshiba Medical Systems, Otawara, Japan). These single-source systems feature a 64 × 0.5 mm detector configuration, automatic tube current modulation, z-axis coverage of up to 32 mm per rotation, and vendor-implemented iterative reconstruction techniques intended to support image quality improvement and radiation dose optimization.

A total of 74 adult patients who underwent lower-extremity CTA were included. Data were retrospectively extracted from archived clinical CT examinations performed across four hospitals (Hospital 1: n = 15; Hospital 2: n = 21; Hospital 3: n = 17; Hospital 4: n = 21). Basic demographic information was obtained from imaging records. No weight or body mass index-based inclusion or exclusion criteria were applied. Mean patient ages across the four hospitals were approximately 52, 64, 64, and 66 years, respectively (Table 1). The study population consisted of 63 males (85.1%) and 11 females (14.9%).

**Table 1:** Patients’ Basic Demographic Characteristics Across Hospitals.

Hospital	No. of Patients	Age years (mean ± SD), range	Female %
1	15	52.3 ± 21.7 (22-81)	1 (6.66%)
2	21	63.5 ± 17.5 (53-80)	8 ((38.1%)
3	17	63.6 ± 13.7 (43-87)	1 (5.88%)
4	21	65.9 ± 13.3 (54-70)	1 (4.76%)

All centres employed a multiphase lower-extremity CTA protocol. Each examination included a localizer/planning scan (normal) followed by a dynamic bolus-tracking sequence (dynamic). A non-contrast helical arterial acquisition was performed selectively based on clinical indication. Contrast-enhanced imaging comprised a primary arterial helical acquisition extending from the abdominal aorta to the ankles (Helical one), followed by a delayed arterial phase acquired in the reverse direction from the ankles to the knees (Helical two), intended to optimize arterial opacification of distal vessels. Contrast arrival was determined using automated bolus tracking, with monitoring scans acquired every two seconds, starting between 10 and 50 seconds after contrast injection.

For each scan phase, the CT systems automatically reported the volume CT dose index (CTDI_(vol)_) and dose-length product (DLP). In addition, cumulative CTDI_(vol)_ and DLP values for the complete examination were recorded for each patient along with total scan length information. Key acquisition parameters, including tube voltage (kVp), tube current (mA, automatically modulated), total scan time, and slice thickness, were documented for all hospitals.

Patient-specific size metrics, including body weight, body mass index (BMI), and size-specific dose estimate (SSDE), were not available for this retrospective dataset. Consequently, dose comparisons in this study reflect differences in protocol design, scan length, phase selection, and acquisition parameters rather than individualized patient radiation dose.

In hospital 4, phase-specific CTDI_(vol)_ and DLP values were not accessible because the CT system was configured to report cumulative dose metrics only. As a result, it was not possible to extract dose contributions from individual scan phases. Hospital 4 was therefore excluded from phase-specific dose analyses but included in the evaluation of total examination dose.

Phase-specific mean CTDI_(__vol)_ and DLP values were calculated by averaging measurements across all patients within each hospital. Descriptive statistics, including mean ± standard deviation and range, were used to compare radiation dose metrics between hospitals and across scan phases.

## Results

A phase-by-phase analysis was performed in three hospitals to isolate the contribution of individual protocol components to overall radiation exposure. This approach was necessary because cumulative DLP alone can mask which acquisition steps disproportionately elevate patient dose. Table 2 and Table 3 show clear phase-specific variation in CTDI_(__vol) _and DLP across three centres.

**Table 2:** Phase-Specific CTDI(vol) (mGy), Mean ± SD (Range). *Dynamic-phase CTDI_(__vol)_ reflects localized dose accumulation during repeated monitoring.

Phase	Hospital 1	Hospital 2	Hospital 3
Normal	16.89 ± 1.76 (14.2-20.5)	27.97 ± 7.63 (14.2-34.2)	18.57 ± 3.59 (14.2-21.3)
Non-contrast	26.6 ± 0 (26.6-26.6)	15.24 ± 2.89 (10.2-22.8)	-
*Dynamic	374.2 ± 488.8 (28.6-719.8)	516.1 ± 186.8 (181.4-904.1)	219.5 ± 118.1 (62.4-406.8)
Helical 1	23.59 ± 6.28 (14.8-31.9)	13.36 ± 1.53 (10.2-14.8)	30.24 ± 12.72 (14.7-39.9)
Helical 2	23.59 ± 6.28 (14.8-31.9)	13.25 ± 1.82 (7.9-14.8)	29.98 ± 13.08 (11.5-39.9)

**Table 3:** Phase-Specific DLP (mGy·cm), Mean ± SD (Range). DLP: dose-length product.

Phase	Hospital 1	Hospital 2	Hospital 3
Normal	3.1 ± 0.42 (2.8-3.4)	5.87 ± 1.42 (2.8-6.8)	3.8 ± 0.73 (2.8-4.3)
Non-contrast	3478.4 ± 150.47 (3372-3584.8)	2038.5 ± 406.3 (1447-2986)	-
Dynamic	56.7 ± 3.25 (54.4-59)	111.35 ± 35.7 (36.3-180.8)	44.63± 24.5 (12.5-81.4)
Helical 1	2766.35 ± 1194.94 (1921.4-3611.3)	1864.19 ± 253.95 (1356.5-2158.7)	4309.69 ± 1568.5 (1995.6-6334.5)
Helical 2	1633.6 ± 841.31 (1038.7-2228.5)	1313.25 ± 220.31 (900.8-1602.4)	2496.33 ± 1124.16 (933.7-4729.7)

For the normal phase, CTDI_(__vol)_ values ranged from 16.89 to 27.97 mGy, with consistently small DLP values (3.23-5.62 mGy·cm), confirming a negligible contribution to the total examination dose.

The non-contrast helical arterial phase was performed in 5 (33.3%) of patients in hospital 1, 18 (85.7%)of patients in hospital 2 and was not required for any patients in hospital 3. This phase was associated with relatively high CTDI_(__vol)_ values of 26.6 mGy and 15.24 mGy, and large DLP values of 3546.9 mGy·cm and 2046.8 mGy·cm in hospitals 1 and 2, respectively. These results indicate that inclusion of the non-contrast helical phase substantially increases cumulative radiation exposure, particularly in hospital 1, where both CTDI_(__vol)_ and DLP were highest.

The dynamic bolus-tracking phase contributed very low DLP values (52.0-105.7 mGy·cm) but showed disproportionately elevated accumulated CTDI₍_vol)_ values (219.5-516.1 mGy) due to repeated axial monitoring at a fixed z-position. Hospital 2 exhibited the highest CTDI_(__vol)_ during this phase, likely reflecting differences in bolus-tracking duration and protocol-defined exposure modulation parameters.

The two contrast-enhanced helical phases together were the dominant contributors to radiation exposure across all centres. Helical phase one yielded DLP values ranging from 1824.2 to 4362.6 mGy·cm, with corresponding CTDI_(__vol)_ values of 13.36-30.24 mGy. Helical phase two produced DLP values of 1286.0-2470.5 mGy·cm, with CTDI_(vol)_ values ranging from 13.25 to 29.98 mGy.

Table 4 summarizes accumulated per-patient parameters for all four hospitals. Scan length and DLP values represent the mean of summed phase-specific values for each patient, with hospital-level averages reflecting the mean total exposure per patient. Although tube voltage (116.7-120 kVp) and slice thickness (3-5 mm) were broadly similar across centres, hospital 4 employed variable tube voltage selection, resulting in a mean kVp of 116.7 ± 7.6, whereas the other hospitals used a fixed tube voltage of 120 kVp. Substantial variation was observed in total mA (170.7-228.5 mA) and total scan length (1921.8-3072.8 mm), pointing to inconsistent anatomical coverage and exposure-modulation practices. The accumulated total DLP (mean of all per-patient totals) varied widely across hospitals.

**Table 4:** Scan Parameters and Total DLP. DLP: dose-length product.

Hospital	kVp	mA	Time (s)	Slice Thickness (mm)	Scan Length (mm)	Total DLP (mGy·cm)
1	120	178.5 ± 11.1	89.3 ± 13.9	5	2695 ± 709	7911.6 ± 2152.2
2	120	170.7 ± 10.4	74.4 ± 11.5	5	3072.8 ± 672.5	5333.2 ± 1199.4
3	120	228.5 ± 44.1	83.4 ± 19.8	5	2034.1 ± 166	6458.45 ± 2684.2
4	116.7 ± 7.6	186.3 ± 16.6	64.7 ± 6.8	3	1921.8 ± 259.5	5754.9 ± 949.4

## Discussion

This multicenter evaluation demonstrated substantial inter-hospital variability in radiation dose for lower-extremity CT angiography (CTA), despite all centers using identical 64-slice CT scanners. These differences were primarily driven by protocol design and acquisition parameter selection rather than scanner hardware. Phase-specific analysis revealed that both non-contrast and contrast-enhanced helical acquisitions were the main contributors to cumulative radiation exposure, with phase-level dose-length product (DLP) values frequently exceeding 1000 mGy·cm.

In hospitals 1 and 2, the non-contrast helical phase produced DLP values exceeding those of the contrast-enhanced arterial acquisition, suggesting that omitting this phase could meaningfully reduce cumulative patient dose. In hospital 3, however, elevated CTDI_(__vol)_ values during the contrast-enhanced helical phases increased DLP, negating any dose-saving benefit of phase omission despite shorter scan lengths. These findings indicate that phase reduction alone is insufficient if tube voltage and automatic tube current modulation are not optimized. Notably, CTDI_(__vol)_ is strongly influenced by both the selected kVp and the behavior of the tube current modulation system, which helps explain the observed inter-hospital variability. Overall, these results underscore the need for a balanced strategy integrating phase selection, exposure parameter optimization, and scan control.

When a non-contrast phase is included, it should be selectively justified for specific clinical indications. Evidence indicates that routine pre-contrast acquisitions offer limited diagnostic benefit in lower-extremity CTA while substantially increasing patient dose [4,5]. Consistent with international recommendations, non-contrast scans should be performed only when clinically indicated and carefully optimized to minimize radiation [1,2]. Practical dose-reduction strategies include limiting scan coverage, using wider collimation, reducing tube voltage, and applying concomitant tube current reductions [1,2].

Although contributing a smaller fraction of total DLP, the bolus-tracking (dynamic) phase imposes a non-negligible radiation burden. Repeated short monitoring scans at a fixed anatomical position result in localized dose accumulation, reflected by elevated accumulated CTDI_(__vol)_ values. Previous studies have shown that excessive bolus-tracking acquisitions increase dose without improving diagnostic yield. Kim et al. demonstrated significant dose reductions by optimizing tube voltage and current during bolus tracking and recommended minimal monitoring settings of approximately 100 kVp/50 mA or 120 kVp/30 mA [10]. These findings contextualize the elevated CTDI_(__vol)_ values observed in hospital 2 and highlight opportunities for targeted optimization without compromising contrast timing accuracy.

Comparison with published literature provides further context. A recent Sudanese study evaluating lower-extremity CTA across 64, 128, and 160-slice CT scanners reported a mean (range) CTDI_(__vol)_ of 7.3 mGy (2.3-22.9 mGy) and a corresponding DLP of ~3000 mGy·cm (279-8374 mGy·cm) [11]. Although formal diagnostic reference levels (DRLs) were not established, these data provide valuable national context. In the present study, mean cumulative DLP values exceeded this reported mean but remained within the upper range, likely reflecting differences in scanner generation and the exclusive use of multiphase protocols on 64-slice systems.

Regional and international comparisons further highlight the magnitude of observed dose levels. Saudi Arabia’s national DRL project reported 75th-percentile values of 9 mGy for CTDI_(__vol)_ and 1040 mGy·cm for DLP for arterial-phase lower-extremity CTA [12], while Egypt reported 37 mGy and 1320 mGy·cm [13]. In Europe, Schegerer et al. suggested a DLP reference of ~1000 mGy·cm [9]. In several centres in this study, DLP for the primary contrast-enhanced helical phase alone exceeded these reference values, even before accounting for additional phases. Similarly, CTDI_(__vol)_ values exceeded Saudi and Egyptian reference levels, indicating suboptimal optimization of exposure parameters, including tube voltage, pitch, and automatic tube current modulation.

High phase-specific DLP values in this study contrast with results from optimization-focused investigations. Kim et al. reported a mean DLP of ~266.6 mGy·cm using an 80 kVp high-pitch protocol [14]. Qi et al. achieved substantial dose reductions using 70 kVp high-pitch CTA combined with iterative reconstruction [15], while Liu et al. demonstrated that 80 kVp CTA with iterative model reconstruction significantly reduced dose without compromising diagnostic confidence [16]. Although these ultra-low-dose protocols primarily employed dual-source CT systems, the differences indicate substantial potential for optimization in current clinical protocols.

Well-established strategies remain effective. Reducing tube voltage from 120 kVp to 70-80 kVp on 64-slice scanners significantly decreases dose while preserving image quality [17]. Prospective studies confirm that 80 kVp protocols improve arterial enhancement and reduce radiation exposure in lower-extremity CTA without compromising image quality [18], demonstrating that simple protocol adjustments are highly effective.

Analysis of cumulative DLP across all scan phases illustrates inter-hospital variability (Table 4). Mean cumulative DLP differed by approximately 1.5-fold between the lowest- and highest-dose centers, reflecting differences in scan length, CTDI_(__vol)_, and phase selection. While reductions in scan length decreased dose in some hospitals, these gains were often offset by higher CTDI_(__vol)_, showing that exposure-related parameters can outweigh anatomical coverage reductions.

Hospital 4, for which only total examination dose data were available, had the shortest mean scan length but a mean cumulative DLP of ~5754.9 mGy·cm, indicating that scan length alone does not determine total radiation burden. Instead, cumulative dose likely reflects combined effects of exposure settings and the number of acquisition phases, consistent with observations from other centres. Elevated CTDI_(vol)_ values can negate the benefits of reduced scan coverage.

Overall, these results highlight the need for harmonized protocol optimization across centres, emphasizing standardization of scan extent, optimization of exposure parameters, and elimination of unnecessary phases. Such measures are essential to achieve consistent dose reduction in lower-extremity CTA within existing scanner capabilities.

Several limitations should be acknowledged. Radiation dose metrics were evaluated without parallel assessment of image quality, limiting conclusions regarding the relationship between dose reduction and diagnostic performance. The number of patients per centre was modest, which may increase uncertainty in hospital-level mean dose estimates. Patient body habitus parameters (weight, height, and body mass index) were not available; therefore, size-specific dose estimates (SSDE) and effective dose could not be calculated, and dose comparisons primarily reflect differences in protocol design, scan length, and acquisition parameters rather than individualized patient radiation dose. Phase-specific dose metrics were unavailable for Hospital 4 due to system reporting constraints, limiting phase-level comparisons for that center. Finally, comparisons with regional and international diagnostic reference levels are constrained by differences in patient populations, protocol definitions, and scanner capabilities.

In summary, elevated radiation doses in the participating centers were mainly due to protocol design rather than scanner limitations. Implementing achievable strategies-such as standardizing scan length, eliminating unnecessary phases, optimizing bolus-tracking parameters, using low-kVp acquisitions where feasible, and consistently applying iterative reconstruction-can substantially reduce dose while maintaining diagnostic quality. These findings also underscore the importance of ongoing staff training and protocol harmonization to ensure consistent application of dose-optimization strategies in routine clinical practice.

## Conclusions

Elevated radiation doses in lower-extremity CTA are predominantly determined by protocol design rather than scanner limitations. Substantial inter-hospital variability underscores the need for standardized protocols that integrate phase selection, scan length optimization, exposure parameter adjustment, and selective use of non-contrast acquisitions. Implementation of low-tube-voltage protocols (70-80 kVp), judicious use of iterative reconstruction, and careful optimization of bolus-tracking parameters represent practical, effective strategies for reducing patient radiation dose while maintaining diagnostic image quality. Harmonized application of these measures across centers is essential to achieve consistent and meaningful dose reduction in clinical practice.
